# Dermoscopic monitoring of pediatric melanocytic nevi regarding pattern and diameter changes^☆^

**DOI:** 10.1016/j.abd.2024.01.004

**Published:** 2024-10-22

**Authors:** Dilara İlhan Erdil, Ayşe Esra Koku Aksu, Vefa Aslı Turgut Erdemir, Duygu Erdil, Cem Leblebici, Asude Kara Polat

**Affiliations:** a Department of Dermatology, University of Health Sciences, İstanbul Training and Research Hospital, İstanbul, Turkey; b Department of Dermatology, Istanbul Medeniyet University, Istanbul, Turkey; c Department of Pathology, University of Health Sciences, İstanbul Training and Research Hospital, İstanbul, Turkey; d Department of Dermatology, Memorial Hospital, Istanbul, Turkey

**Keywords:** Nevus, Pigmented, Dermatoscopy, Child, Neoplasms, Skin

## Abstract

**Background:**

Childhood and adolescence are dynamic period in terms of nevogenesis, and the development and growth of new melanocytic nevus are frequently observed. In this study, the aim was to examine the pattern and diameter changes seen in the follow-up of pediatric melanocytic nevus.

**Objectives:**

To describe the pattern and diameter changes seen in the follow-up of pediatric melanocytic.

**Methods:**

Our study involved the assessment of 301 pediatric melanocytic nevi in 50 patients attended at the Dermatology Clinic of Istanbul Training and Research Hospital between January 2008 and 2022. The pediatric melanocytic nevi were diagnosed clinically and dermoscopically. Subsequently, we conducted video-dermoscopic monitoring of these nevi over a span of 3 months to 3 years.

**Results:**

46% of our patients were female (n = 23), with a mean age of 11.5 years. While the pattern of nevi was globular in 40% patients, the rate of globular pattern decreased to 30% in the follow-up. The basal homogeneous nevus pattern was seen in 10% patients, but was detected in 13.9% in the follow-up. Peripheral globules were observed in 19.3% of the cases, but in the follow-up, 61.1% of the globules regressed completely. Nevus excision was indicated in only 11 of 301 nevi.

**Study limitations:**

Single-center study and a small of studies available on this subject.

**Conclusions:**

Pediatric melanocytic nevi can show dynamic changes compared to nevi in adults. In this study, growth rates, dermoscopic features, and pattern changes seen in the follow-up of melanocytic nevi were evaluated. The globular pattern was observed most frequently. The presence of peripheral globules is frequently observed in pediatric melanocytic nevi with regression during the follow-up period.

## Introduction

Childhood and adolescence are dynamic processes in terms of nevogenesis, and the development and growth of new melanocytic nevus are frequently observed.^1^ Melanomas, although rare, can also be seen in the pediatric age group.^2^ While routine monitoring of nevi in the pediatric age group is not recommended unless there is a suspected condition, detecting the characteristics of benign lesions and their growth patterns is valuable for distinguishing them from malignancies.^3^

In the pediatric age group, changes are frequent because it is a dynamic process, and alterations that cause melanoma suspicion in adults can be expected in the pediatric age group. It is known that there is a variation in nevus diameters, numbers and patterns within age groups.^4^ Due to the limited number of studies in the literature on dermoscopic monitoring of pediatric nevi, our study aimed to show the dynamic pattern and diameter modifications in pediatric nevi.

## Method

In our study, patients who applied to the Dermatology Clinic of Istanbul Training and Research Hospital between January 2008‒2022 and diagnosed with pediatric melanocytic nevus clinically, and dermoscopically were included. Patients who had recorded at least twice with video-dermoscopy every three months were included. In the study, 301 pediatric melanocytic nevi belonging to 50 patients were evaluated.

Dermoscopic records of the subjects included in the study were taken with FotoFinderdermoscope (FotoFinder Systems GmbH, Bad Birnbach, Germany).

The study was approved by the Local Ethics Committee. Patient files and photographs were retrospectively reviewed through medical records of the hospital and videodermoscopy database. The patients in this manuscript have given written informed consent to the publication of their case details.

The following characteristics were recorded in a database specifically designed for the study: demographic findings of the patients, nevus localization, nevus pattern (baseline and follow-up), nevus diameter (baseline and follow-up at 3/12/36^th^ months), and histopathological results of nevi that underwent total excision were recorded. The change in nevus diameter during follow-up was measured in millimeters based on the longest axis. The presence of peripheral globules, the outcome of peripheral globules, and the characteristics of peripheral globules were recorded. Characteristics of peripheral globules were evaluated according to being around the entire lesion (circumferential) versus focal presence, typical (uniform shape and colors), and regular (single rim of globules or multiple rims of globules).

### Statistical analysis

Statistical analyses were performed with SPSS version 23.0 program. The conformity of the variables to the normal distribution was examined by histogram graphs and Kolmogorov-Smirnov/Shapiro-Wilk test. Mean, standard deviation, median, minimum, and maximum values were used while presenting descriptive analyses. The Mann-Whitney *U* Test was used when evaluating non-normally distributed (nonparametric) variables between two groups, and the Kruskal Wallis Test was used when evaluating between more than two groups. The Bonferroni, multiple comparison test was used for the significant difference between the groups. While presenting the categorical variables, the frequency and percentage values of the variables were used; p-value below 0.05 was considered a statistically significant result.

## Results

**Figure 1:**
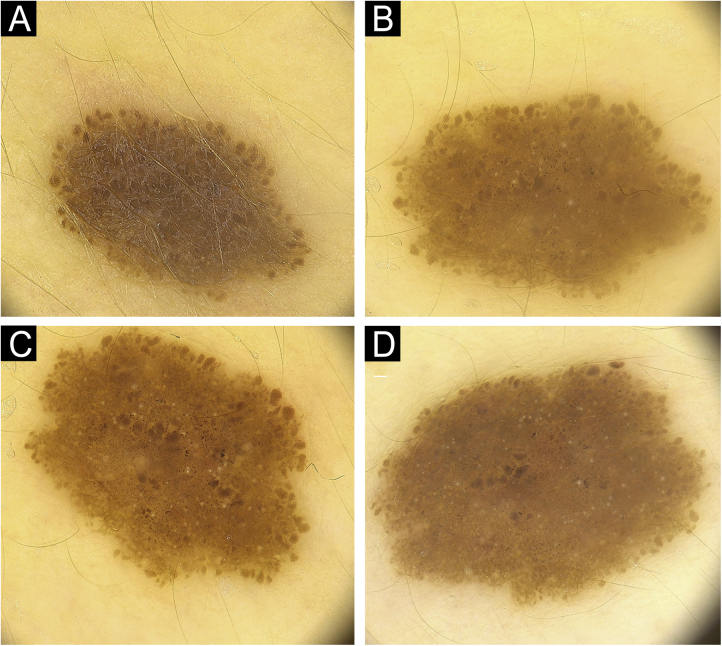
(A) Pediatric melanocytic nevus with a diameter of 3.5 mm homogenous-globular pattern in the center and peripheral globules arranged regularly in a single row. (B) There is an increase in nevus diameter to 4.3 mm in 12 months with similar number of peripheral globules. (C) Increase in nevus diameter continues, with a decrease in peripheral globules. (D) Noticeable enhancement in the central homogenous structure and a significant decrease in peripheral globules, nevus starts to stabilize with a diameter of 4.6 mm at 36^th^ months.

**Figure 2:**
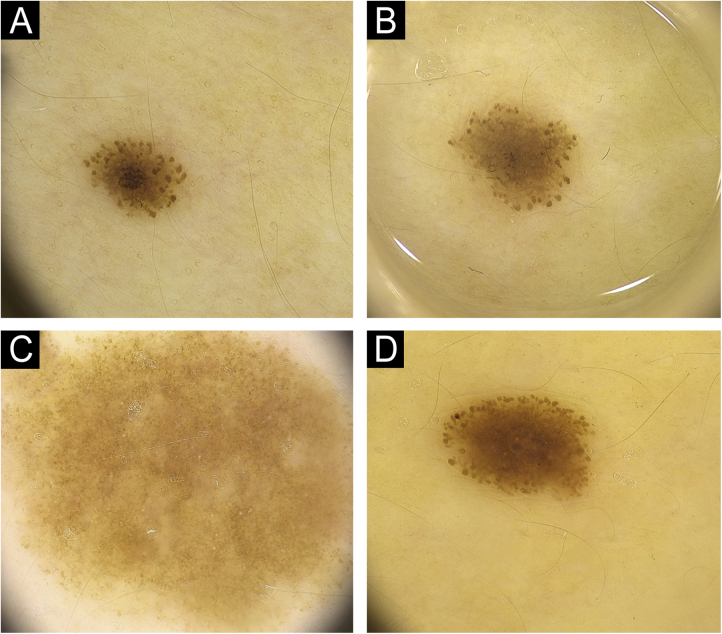
(A) Pediatric nevus with a diameter of 2.3 mm with darker globular-homogenous pattern and the globules are prominent at the periphery. (B) In the follow up of the same nevus, diameter increases to 3.5 mm in 12 months, homogenization in the central region and a lightening of color to light brown are observed. (C) In a different nevus, a globular structure is vaguely discernible around and the nevus has taken an almost homogenous structure with stabilization. (D) Nevus with a raised dermal component in the center and a continuation of the globular structure at the periphery.

Fifty patients with a total of 301 nevi were included in the study. Patient demographic, clinical, and histopathological characteristics are shown in Table 1. 46% of our patients are female (n = 23), with an average age of 11.5 years. In 90% of the cases, trunk and extremity involvement, 6% palmoplantar involvement, and 4% scalp/face involvement were observed. The globular pattern was 40% at baseline and decreased to 30% at follow-up. A reticular pattern was detected at a rate of 31%, and no change was observed in the follow-up. Basal homogeneous nevus pattern was 10%, it was detected as 13.9% in the follow-up. The homogeneous/homogeneous reticular/homogeneous globular nevus pattern ratio was 18.1% at baseline and increased to 27.3% at follow-up. No significant difference was observed in the proportions of parallel groove, fibrillar, and reticular patterns. There was no significant difference in the size of the nevi by localization (p = 0.6). The presence of peripheral globules was observed in 19.3% of the cases. In the follow-up, 61.1% of the globules completely regressed, 13% decreased, and 5.6% increased. No change was observed in the number of peripheral globules during the follow-up period in 20.4% of the cases. The increase in nevus diameter over time, accompanied by a decrease in peripheral globules and finally, the stabilization phase of the nevus is shown in Fig. 1. In Fig. 2, homogenization in the pattern of nevi transitioning into the stabilization phase, as well as color fading, is shown. Along with stabilization, there is a reduction in globular structure and accentuation of the dermal component in some nevi.

**Table 1:** Nevi clinical characteristics in the follow up period.

		n	%
**Gender**	Female	23	(46.00)
	Male	27	(54.00)
**Localization**	Trunk and extremity	270	(89.70)
	Acral	17	(5.65)
	Scalp/face	14	(4.65)
**Pattern (baseline)**	Fibrilar	7	(2.35)
	Globular	119	(39.93)
	Globular + Homogenous	13	(4.36)
	Globular + Reticular	13	(4.36)
	Homogenous	30	(10.07)
	Homogenous + Reticular	11	(3.69)
	Parallel furrow	13	(4.36)
	Reticular	92	(30.87)
**Pattern (follow-up)**	Fibrilar	7	(2.36)
	Globular	89	(30.07)
	Globular + Homogenous	21	(7.09)
	Globular + Reticular	14	(4.73)
	Homogenous	41	(13.85)
	Homogenous + Reticular	19	(6.42)
	Paralel furrow	13	(4.39)
	Reticular	92	(31.08)
**Histopathology**	Compound nevus	2	(18.8)
	Dysplastic nevus	6	(54.5)
	Junctional nevus	2	(18.8)
	Spitz nevus	1	(9.09)
**Presence of peripheral globules**	Present	58	(19.27)
	Absent	243	(80.73)
**Characteristics of peripheral globules**	Circumferential	50	(86.2)
	Regular	54	(93.1)
	Typical	55	(94.8)
**Peripheral globule outcome**	Disappeared	33	(61.11)
	No change	11	(20.37)
	Decreased	7	(12.96)
	Increased	3	(5.56)

Peripheral globules (n = 58) were observed in 86% circumferential, 93% regular, and 94% typical characteristics. Nevus excision was required in only 11 of 301 nevi, and the excised nevi were diagnosed as dysplastic nevi (n = 6), compound (n = 2), junctional (n = 2) and Spitz nevi (n = 1). The average age according to the pattern is summarized in Table 2. There was no difference in diameter change according to the presence of peripheral globules (p = 0.1), summarized in Table 3.

**Table 2:** Nevi pattern according to average age.

		Age		
		Avg.	Std. dev.	Median
**Pattern**	Fibrilar	8.86	±4.60	8.00
	Globular	12.75	±3.38	13.00
	Globular + Homogenous	16.23	±1.69	17.00
	Globular + Reticular	13.62	±3.99	14.00
	Homogenous	13.63	±2.95	14.00
	Homogenous + Reticular	14.82	±2.14	15.00
	Parallel Groove	9.92	±4.94	10.00
	Reticular	14.16	±2.79	14.00

**Table 3:** Nevi size according to presence of peripheral globules.

	Presence of concomitant peripheral symmetric globules						
	None			Peripheral globules			p
	Avg.	Std. dev.	Median	Avg.	Std. dev.	Median	
**Baseline**	4.40	±2.05	4.00	4.39	±1.95	4.10	0.940
**3-month**	4.60	±1.97	4.20	4.73	±1.89	4.40	0.582
**12-month**	4.67	±2.10	4.25	5.12	±2.03	4.70	0.115
**36-month**	5.20	±2.17	4.60	5.93	±2.03	6.15	0.112

Mann Whitney *U* Test.

## Conclusion

In this study, growth rates, dermoscopic features, and pattern changes in the follow-up period of pediatric nevi were evaluated. Pediatric melanocytic nevi may show dynamic changes compared to adult nevi, and the pattern changes reported in the literature are variable. Globular pattern was most frequently observed in our study in the pediatric age group. Globular pattern is the predominant pattern in children and adolescents, and it has been reported that the reticular pattern increases with age.^1^, ^2^ Number of globular nevi decreases with age, up to 1% in advanced age (>75).^2^

It is thought that globular nevi persist but acquire dermal features and acquire a homogeneous appearance.^1^ In our study, we found that the globular pattern rate, which was 40%, decreased to 30% over time. We found that the globular pattern changed towards a homogeneous/homogeneous globular pattern, as the other patterns remained mostly constant.

There are many studies and concepts related to nevogenesis. Because the globular and reticular patterns show age-related changes, nevogenesis was thought to occur through different pathways. First pathway demonstrates that nevi in the globular pattern develop within large dermal nests in childhood, which are thought to maintain their general pattern as the child grows. The second nevogenesis pathway is the development of epidermal melanocytes in the reticular pattern, which is acquired in adulthood and is caused by external factors such as intermittent UV exposure. From a histopathological point of view globular nevi appear in childhood and are usually in the compound or dermal component. Reticular nevi show junctional development.^4^

Although a transition from globular to reticular pattern was mentioned in various studies, such pattern change was not apparent in our study.^5^, ^6^ Longer follow-up times may be required for enhanced reticular patterning due to UV damage. There is a hypothesis that globular nevi evolve dermoscopically in two ways, first one is progression into intradermal nevus. This condition may be accompanied by a papillomatous or structureless appearance and nevus may evolve into a lighter color. The second one is evolution towards the mixed pattern. It can be structureless accompanied by reticular lines or it can be in the form of a fried egg appearance.^7^

There are studies in which dermoscopic classification is evaluated by histopathological correlation using longitudinal Reflectance Confocal Microscopy (RCM). In these studies, the idea of nevogenesis occurring via two pathways was supported. However, no significant changes were observed in nevi with RCM during the follow-up, and the rare changes observed were also in the same compartment (epidermal or junctional and dermal). This situation challenged the paradigms of downward or upward migration of melanocytes.^7^, ^8^, ^9^ On the other hand, this study was performed in adults and further studies are needed to show the evolution of nevi with RCM in the pediatric age group where the main dynamic changes are present. Thus, the correspondence of these dynamic dermoscopic changes that we see in the pediatric age group with RCM will elucidate the nevus evolution.

The presence of peripheral globules is frequently observed in pediatric nevi and was present in 19% of our cases. Peripheral globule ratios peak in the adolescent age group and then decline rapidly. During the follow-up period, regression of peripheral globules was observed in most of our cases. Peripheral globules are an expected finding in the pediatric age group and generally do not require additional intervention. However, after the age of 30, close digital dermoscopic follow-up is recommended even in the absence of other melanoma-specific criteria.^1^ Accompanying melanoma specific criterias such as blotch, atypical dots and globules, or atypical vessels with the presence of peripheral globules has the greatest risk of melanoma.^10^

In our study, peripheral globules were often circumferential, regular and typical. Peripheral globules accompanying the nevus have been reported to be typical, circumferential. Atypical, asymmetrical peripheral globule distribution supports melanoma especially in the adult age group.^11^ Melanoma was not detected in the presence of peripheral globules in any of our cases even with the presence of atypical globule appearance. Although peripheral globule is associated with enlargement in nevi, we did not observe a significant change in diameter according to the presence of peripheral globules.^12^ Since in the pediatric age group all nevi can enlarge with or without peripheral globules, there might be no significant growth change according to peripheral globule presence.

In conclusion, most common patterns were globular and reticular patterns with decreasing order in the pediatric period in our study. We observed that the globular pattern changed towards homogeneous/homogeneous globular pattern in the follow-up. Although peripheral globules often accompany, they can be considered as a benign finding in this age group. Knowing the benign features and the pattern changes will help to differentiate the benign lesion from the malignant lesion. Pediatric age group studies supported by RCM are needed to elucidate nevogenesis.

## Financial support

None declared.

## Authors’ contributions

Dilara Ilhan Erdil: Substantial contributions to conception and design, acquisition of data, analysis and interpretation of data, involved in drafting the manuscript, critical review of important intellectual content, critical review of the literature, final approval of the version to be published, ensured that questions related to the accuracy or integrity of any part of the work are appropriately investigated and resolved.

Ayşe Esra Koku Aksu: Substantial contributions to conception and design, acquisition of data, analysis and interpretation of data, involved in drafting the manuscript, critical review of important intellectual content, critical review of the literature, final approval of the version to be published, ensured that questions related to the accuracy or integrity of any part of the work are appropriately investigated and resolved.

Aslı Vefa Erdemir: Acquisition of data, analysis and interpretation of data, involved in drafting the manuscript, final approval of the version to be published.

Duygu Erdil: Acquisition of data, analysis and interpretation of data, involved in drafting the manuscript, final approval of the version to be published.

Cem Leblebici: Acquisition of data, analysis and interpretation of data, involved in drafting the manuscript, final approval of the version to be published.

Asude Kara Polat: Acquisition of data, analysis and interpretation of data, involved in drafting the manuscript, final approval of the version to be published.

## Conflicts of interest

None declared.

## References

[1] Oliveria S.A., Geller A.C., Dusza S.W., Marghoob A.A., Sachs D., Weinstock M.A. (2004). The Framingham school nevus study: a pilot study. Arch Dermatol.

[2] Zalaudek I., Schmid K., Marghoob A.A., Scope A., Manzo M., Moscarella E. (2011). Frequency of dermoscopic nevus subtypes by age and body site: a cross-sectional study. Arch Dermatol.

[3] Scope A., Marchetti M.A., Marghoob A.A., Dusza S.W., Geller A.C., Satagopan J.M. (2016). The study of nevi in children: principles learned and implications for melanoma diagnosis. J Am Acad Dermatol.

[4] Zalaudek I., Catricala C., Moscarella E., Argenziano G. (2011). What dermoscopy tells us about nevogenesis. J Dermatol.

[5] Fortina A.B., Zattra E., Bernardini B., Alaibac M., Peserico A. (2012). Dermoscopic changes in melanocytic naevi in children during digital follow-up. Acta Derm Venereol.

[6] Cengiz F.P., Yilmaz Y., Emiroglu N., Onsun N. (2019). Dermoscopic evolution of pediatric nevi. Ann Dermatol.

[7] Woltsche N., Schmid-Zalaudek K., Deinlein T., Rammel K., Hofmann-Wellenhof R., Zalaudek I. (2017). Abundance of the benign melanocytic universe: dermoscopic-histopathological correlation in nevi. J Dermatol.

[8] Pellacani G., Scope A., Farnetani F., Casaretta G., Zalaudek I., Moscarella E. (2014). Towards an in vivo morphologic classification of melanocytic nevi. J Eur Acad Dermatol Venereol.

[9] Pellacani G., Scope A., Ferrari B., Pupelli G., Bassoli S., Longo C. (2009). New insights into nevogenesis: in vivo characterization and follow-up of melanocytic nevi by reflectance confocal microscopy. J Am Acad Dermatol.

[10] Moraes A.F.A., Blumetti T.C.M.P., Pinto C., Bertolli E., Rezze G., Marghoob A.A. (2022). Melanoma with peripheral globules: clinical and dermatoscopic features. J Am Acad Dermatol.

[11] Reiter O., Chousakos E., Kurtansky N., Nanda J.K., Dusza S.W., Marchetti M.A. (2021). Association between the dermoscopic morphology of peripheral globules and melanocytic lesion diagnosis. J Eur Acad Dermatol Venereol.

[12] Pampin-Franco A., Gamo-Villegas R., Floristan-Muruzabal U., Pinedo-Moraleda F.J., Perez-Fernandez E., Lopez-Estebaranz J.L. (2021). Melanocytic lesions with peripheral globules: results of an observational prospective study in 154 high-risk melanoma patients under digital dermoscopy follow-up evaluated with reflectance confocal microscopy. J Eur Acad Dermatol Venereol.

